# Comparison of the Properties of Alkali-Phenolic Binder in Terms of Selection of Molding Sand for Steel Castings

**DOI:** 10.3390/ma12223705

**Published:** 2019-11-10

**Authors:** Mariusz Łucarz, Dariusz Drożyński, Jan Jezierski, Andrzej Kaczmarczyk

**Affiliations:** 1 Faculty of Foundry Engineering, AGH University of Science and Technology, 30-059 Kraków, Poland; eumar@agh.edu.pl (M.Ł.); dd@agh.edu.pl (D.D.); 2 Department of Foundry Engineering, Silesian University of Technology, 44-100 Gliwice, Poland; 3 Pioma-Odlewnia Sp. z o.o., 97-300 Piotrków Trybunalski, Poland; andrzej.kaczmarczyk@pgosa.pl

**Keywords:** alkali-phenolic binder, casting mold, molding sand properties, thermal analysis

## Abstract

This article presents the results of experiments related to the process of replacement of the currently used furfuryl resin molding sand technology with a new alkali-phenolic technology. The new binder is characterized by a set of technological advantages and is considered more ecological compared to the furfuryl resin. However, the molding sand produced on the basis of the alkali-phenolic resin features lower strength compared to the sands containing furfuryl resin. This article presents a comparative study of sands made using various alkali-phenolic binders, aimed at the selection of a resin with strength parameters and other features that are both desirable and useful for new technology applied in a foundry.

## 1. Introduction

The alkali-phenolic process is a binary bonding system, consisting of a water reactor and a liquid ester as a reaction partner. The molding sand produced in this process utilizes alkali-phenolic resins cured with an organic ester (alcohol-based). The phenolic resoles used in the system are prepared by reacting 30–55% formaldehyde solution with a lesser amount of phenol, using a strong alkali catalyst such as sodium or potassium hydroxide, at temperatures below 110 °C (pH > 7) [1]. The vast selection of hardeners allows for production of various mixtures with different curing times, which is one of the most important parameters of resin-bonded molding sand. The most important advantages of the so-called Alphaset system (also known as ester-cured alkaline phenolic no-bake binder system) are: low odor, virtually no smoke, the ease of stripping, good finishing, low veining, minimal erosion and very good hot strength [1].

The alkali-phenolic process is typically used for small- and medium-size castings. However, it was proven that the process can be also successfully used for large-size steel castings (which are the domain of the foundry plant analyzed in the presented research) [2]. It can be used for any casting alloy. The lack of nitrogen and sulfur among the resin-polymerizing substances is beneficial for steel- and ductile-iron-castings production.

Depending on the manufacturer of the resin for the alkali-phenolic process, 1 to 2 weight parts of highly-alkalic phenolic resin (resol-type) are added to the grain matrix, followed by aliphatic ester in the ratio of 18% to 25% in relation to the resin. When chromite or olivine sand is used, a greater resin quantity is added, i.e., about 2 weight parts and 20% of the ester mass (with a resin/hardener ratio of 5/1). The chemical reactions between specific esters or their mixtures with the resin containing potassium hydroxide lead to curing and solidification. Depending on the ester used, the curing time can be adjusted, typically to between 5 and 30 min [3,4,5].

The main attribute of the alkali-phenolic process is that a two-step curing process occurs during creation of the chemical bonds. At ambient temperature, an initial (partial) bonding (binder polymerization) occurs, which provides the core mass (or the molding sand) with strength sufficient for manipulation, ensuring thermoplasticity of the prepared sand in medium temperatures. This thermoplasticity compensates for the thermal expansion of the sand grains, which, in turn, nearly completely eliminates cracking of casting molds and molten-alloy leakage problems. Heat produced by the liquid metal (casting) finishes the resin solidification process. Therefore, this technology guarantees dimensional stability and resistance to erosion of liquid metal [6,7].

The alkali-phenolic system, widely known as the Alphaset process, is widely used in foreign foundry plants, while in Poland, it is still not as popular. For example, it was approved as an industrial standard for the production of steel castings in England [8]. All of the aforementioned advantages suggest that this technology is the most suitable and environmentally friendly among those proposed for the foundry industry. Its benefits are most notable in steel foundries, as it significantly improves casting quality and reduces costs associated with their treatment. Additionally, the total volume of hazardous substances (mostly gases) generated when the binding system is in use is much lower than other resin-based systems [9]. Further experiments on the gas emission of commercial Alphaset binders proved the same—their quality, from that point of view, is better than the case of other competitive substances, but they vary substantially among different suppliers [10,11]. However, there is a substantial problem in this system concerning the reclamation process of the molding sand used, which means that particular methods of reclamation have to be used in order to ensure that the reused sand meets quality requirements [12].

There are only a few articles presenting the results of a comparison between the advantages and disadvantages of the alkali-phenolic and furan processes. One of them concludes that the choice between two binders (alkali-phenolic and furan) for green field foundries depends on many factors, the main ones being: the metal to be cast, the compatibility with cheap and available sand, and the investment capability for the mechanical and thermal reclaimer. Moreover, the authors claim in the same article that modern Alphaset and furan formulations are capable of working as clean binder systems at an optimum addition level with reclamation, still meeting the functional requirements of modern foundries [12]. In terms of reclamation, in order to achieve the best results, it is crucial to apply a two-stage reclamation process. It should consist of good quality mechanical reclamation and then, for the best results, a thermal reclamation stage. After the first stage, the rate of reclamation can be as high as 60–80%, but it is crucial to use sodium-based resin to bind this reused sand matrix. After the thermal reclamation, the fresh sand can be nearly 100% substituted by reclaimed material. However, it is important to use special additives (0.6–1.0%) to the shakeout sand just prior to its entry into a thermal reclaimer, in order to make this possible [1]. Another important issue is the utilization of the after-reclamation dust, which was already discussed by the authors in [13]. They analyzed several molding sand compositions, using different binding reagents, including the ones used in the Alphaset process. The results proved their suitability for modern reclamation processes. The importance of reclamation and waste management was explained by the authors in their work about the Swedish approach to the problem of foundry wastes [14]. They presented the vast experience of their foundry industry and focused on the efficient re-use of solid wastes, including a reclaimed sand matrix.

In [15], the authors present the results of the tests of the mechanical properties of molding sand bonded by Alphaset resin, proving its good quality. The authors of [2] present the analysis of various binding systems in terms of large-size castings, concluding that the development of optimal molding sands for the production of castings, taking into account the restrictive requirements for environmental protection, is a very current issue from the point of view of the structure of the European foundry industry. They claim that Alphaset strength properties are good enough to be attractive for foundries [2].

This article presents a case study (both in terms of experimental laboratory and industrial tasks) that describes one of the Polish steel foundries. The main goal was to check the possibility of replacement of the old furan resin technology with the alkali-phenolic one, based on the chemicals present on the domestic market. Different mixtures of molding sand were examined and the recommendations were formulated for the specific working conditions and requirements of the foundry.

## 2. Materials and Methods

The experiments were conducted on four binders, labelled A1, A2, A3 and A4, with the use of four hardeners, labelled 1, 2, 3 and 4. The sand matrix was quartz sand of two grades:

- SIBELCO (S)—material widely used on the domestic market for the sieve analysis parameters (0.2/0.32/0.16), mean grain size d_L_ = 0.25 mm, uniformity index 85% (J85) and surface area of 8.75 m^2^/kg.
- Circular sand from the analyzed Pioma steel foundry (P) of the grain size parameters (0.4/0.2/0.32), mean grain size d_L_ = 0.33 mm, uniformity index 91% (J91) and surface area of 6.22 m^2^/kg.

Table 1 shows the molding sand mixtures used during the experiments. The same experimental plan was repeated twice, for both S and P sand types. Market names of the materials were given, as this was the basis for the selection of resin and hardeners. Each name gives specific properties which are crucial from the point of view of specific foundries. This article does not recommend any system or suggest the best one. The selection is always the matter of an optimization process, based on several factors, including specific foundry conditions.

## 3. Preparation of Materials and Methodology

Thermal analysis of the alkali-phenolic binders was conducted in order to obtain necessary data and full knowledge of their properties. The laboratory furnace was heated at a rate of 10 °C/min up to 1000 °C. At this temperature, all organic compounds should fully degrade or simply burn out. The samples were prepared by mixing the resin with the hardener in a ratio of 1 wt.% part of the resin and 25% of hardener in relation to the resin amount. After 24 h, the solid binder was crushed and prepared for thermal analysis. All samples were prepared in a LM-1 laboratory rotary mixer, supplied by the Foundry Institute in Kraków, Poland. At first, the weighed portion of the sand matrix was loaded into the mixer, and then the hardener was added. After 90 s of mixing, the resin was dosed, and then another mixing occurred for a further 90 s. The prepared mixture was poured into small standard sampling molds, which are used for measuring the properties of molding sand. The molds were mounted on the LUZ-1 type vibration compacting device, supplied by WADAP Wadowice, Poland, which was switched on for 15 s with an amplitude with maximum vibrations of 2 mm. After the compaction, the upper element was taken off, the excess of the sand was cut off and the essential part of the sample shaping mold was disassembled. The shaped samples were left for final curing under ambient conditions. Sets of standard 8-shape samples for R^u^_m_ tensile strength testing_,_ longitudinal samples for R^u^_g_ bending strength testing, and cylindrical samples for P^u^ permeability and S abrasive resistance (abrasion) testing were prepared.

The R^u^_m_ tensile strength and R^u^_g_ bending strength of the cured samples were measured in accordance with PN-83/H-11073 Polish standard [16]. The tests were conducted on a universal apparatus used to measure the mechanical properties of molding sand of LRu-2e type, supplied by Multiserw-Morek, Wadowice, Poland, for three curing times after the compaction: 1, 3, and 24 h. For each curing time, measurements were carried out for the three samples and the presented results are the average of these results.

The P^u^ permeability in the cured state was measured in accordance with PN-80/H-11072 Polish standard [17]. The tests were carried out on the LPiR-1 type apparatus manufactured by WADAP, Wadowice, Poland. The cured cylindrical sample was mounted inside a small sleeve with a pressurized rubber seal inside, tightened to the sample side using compressed air and pressurized by a small manually driven pump. The permeability of each molding sand was determined for the three samples after a 24 h curing period and the results were presented in the graph as an average value.

The abrasion resistance of each molding sand was determined in accordance with BN-77/4024-02 trade standard [18]. The measurements were carried out on apparatus manufactured by Stalowa Wola Steelworks (HSW), Stalowa Wola, Poland. The single experiment consisted of mounting a weighed sand sample on the mounting device (after a 24 h curing period). Then, the sample was subjected to electrically driven rotation of 1 revolution per second. During this rotary movement, a steel shot of 1 mm diameter fell onto the sample from a height of 307 mm. A total of 1750 g of steel shot was used, weighed with 1 g accuracy. Measurements were conducted on three samples and the average value was calculated and presented. All experiments were carried out at ambient temperature T_ot_ = 17.5–21.5 °C and relative humidity W_w_ = 45%–55%.

**Figure 1:**
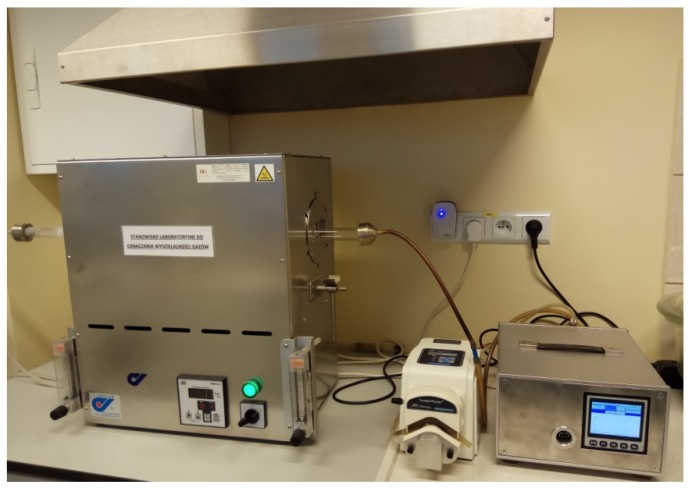
Gas emissivity measuring device.

The gas emissivity test for the sand samples was carried out on the research stand presented in Figure 1, equipped with a PRC 30M/1300 pipe-type furnace by CZYLOK, Jastrzębie – Zdrój, Poland, a peristaltic BT100-2J pump by Longer Pump and a control system.

The single measurement consisted of heating the pipe-type furnace (quartz pipe) up to 1000 °C, then inserting a small ceramic pot containing 0.2 g (for the resin examination) and 1 or 3 g (for the molding sand used) samples into it, out of the heating zone. The samples were prepared with ±0.001 g accuracy. The results of the conducted measurements were converted into one gram of sample mass. The curves in the figures represent the average values of the gas emissivity tests. The resins tested in this part of the experiment had the compositions given above in Table 1. The sand samples were cut from the samples used for the mechanical properties evaluation. After the sample was introduced, one side of the pipe was sealed and the second was connected to the peristaltic pump in order to produce a vacuum inside the reactor to suck out the emitted gases. Then, the sample was inserted into the heating zone, where rapid heating up to the test temperature was carried out and the emitted gas volume was measured.

## 4. Results

**Figure 2:**
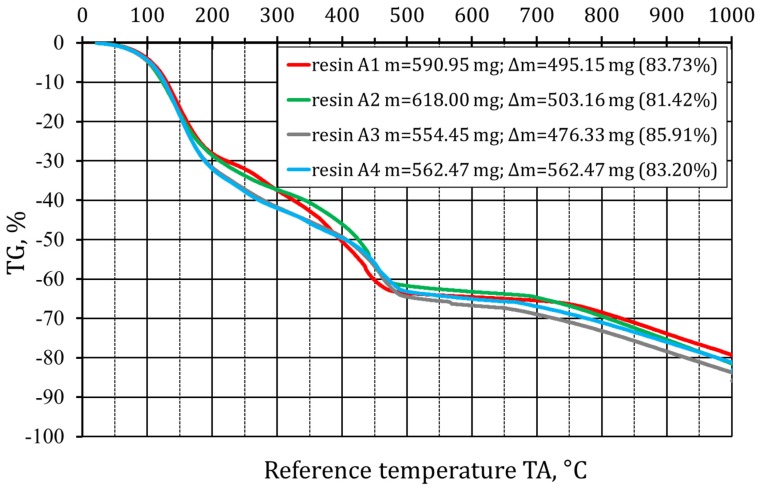
Thermal analysis of the tested alkali-phenolic binders.

Figure 2 presents the thermal analysis results for the analyzed binders. As can be seen, their degradation and thermal destruction process is similar. The temperatures of 150 °C, 450 °C and over 750 °C may be indicated as the ones in which mass changes of the analyzed binders may be observed. Unlike in the case of other binders (for example, furfuryl), the following stages of the binder degradation and destruction are visible, which provide the alkali-phenolic binder with specific grain matrix expansion compensation features, while maintaining the appropriate strength properties [19,20]. The temperature graphs (TG curve) show two distinct features at temperatures around 150 °C and 450 °C for all tested binders. The first feature at 150 °C probably indicates thermoplasticity of the alkali-phenolic binders, reported in the literature, i.e., thermal susceptibility of the sand (partial binder degradation), but without the loss of the binding force. At the second temperature, i.e., over 450 °C, the organic materials are partially burnt, thus creating a space in the mold for the expanding sand matrix as a result of the polymorphic transformation. Only over 750 °C does the next binder destruction stage take place. At a temperature of 1000 °C, a full binder burning process does not occur due to its non-organic compounds content (sodium or potassium—the alkali-phenolic binder ingredients).

**Figure 3:**
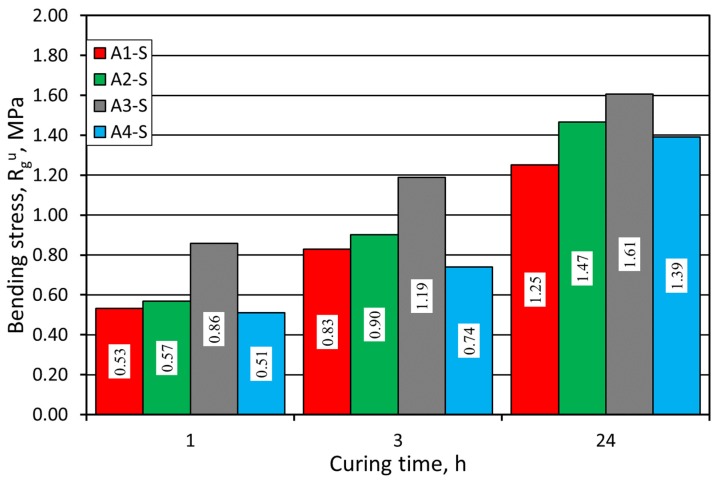
Molding sand R^u^_g_ bending stress test carried out on SIBELCO (S) grain matrix.

Figure 3 shows the results of the R^u^_g_ bending stress test of the molding sands, performed on SIBELCO (S) grain matrix with various alkali-phenolic binders. In terms of bending strength, the A3 binder is by far the best. The strength value was increasing over the curing time, and the following results were achieved after 24 h (from the best to the worst): A3 > A2 > A4 > A1.

**Figure 4:**
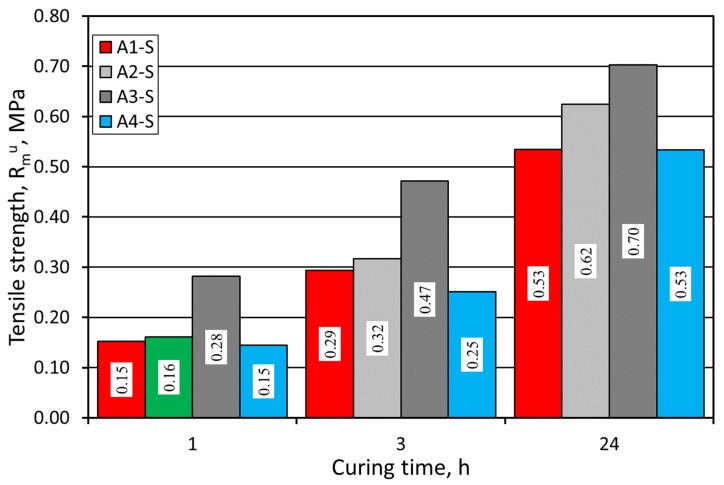
Molding sand R^u^_m_ tensile strength test carried out on SIBELCO (S) grain matrix.

To estimate the mechanical properties of the molding sands with alkali-phenolic binders, R^u^_m_ tensile tests in the cured state were also performed. The results are presented in Figure 4. Also, for this parameter, the molding sand with A3 binder gave the best results.

**Figure 5:**
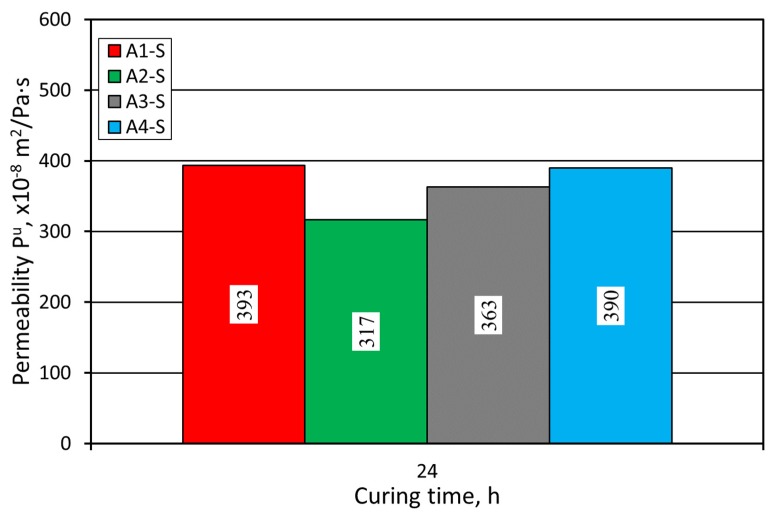
Molding sand P^u^ permeability test carried out on SIBELCO (S) grain matrix.

To ensure sound casting production, it is important to provide a proper dissipation of the gas emitted during the mold pouring process. The sand permeability is the parameter used to compare the conditions of gas flow created when using a particular sand matrix and binder. Figure 5 presents the results of P^u^ permeability test. Due to the use of the same grain matrix and binder volume, the analyzed sands are characterized by similar permeability.

**Figure 6:**
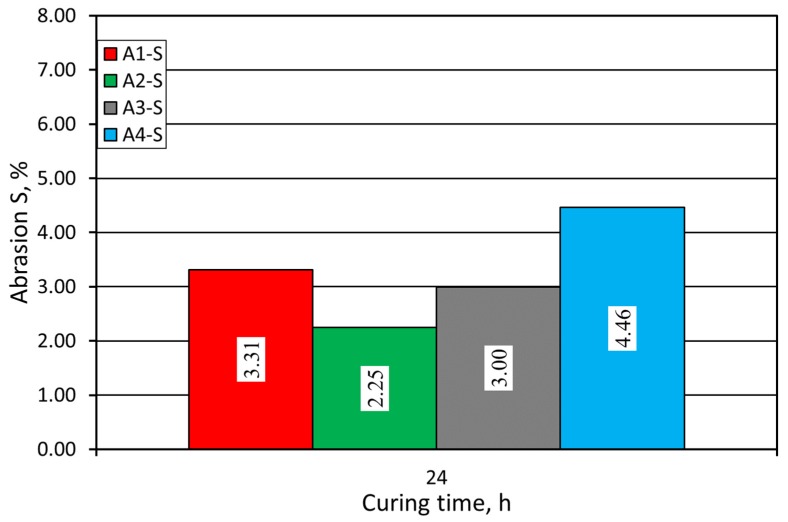
Molding sand S abrasion test carried out on SIBELCO (S) grain matrix.

Another important quality parameter of the molding sand is its S abrasion (abrasive resistance). The results of the evaluation of this parameter are presented in Figure 6. In this case, the sand with A2 binder was the best, followed by the sand with A3 binder. This is associated with a general rule in which sands with higher strength are characterized by better abrasion.

**Figure 7:**
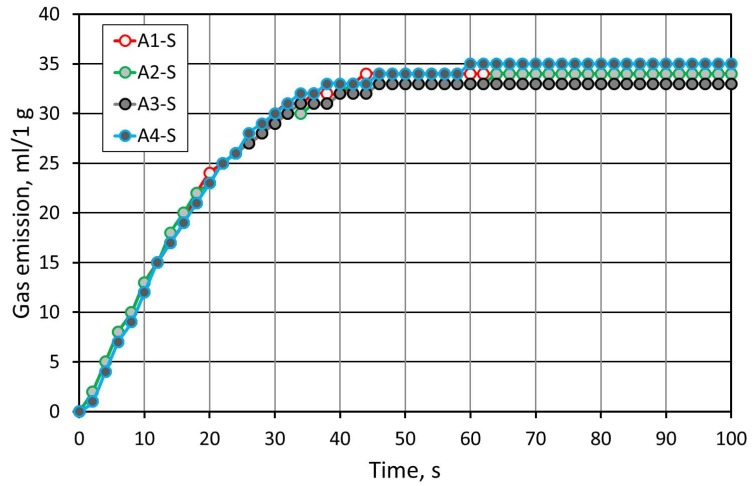
Comparison of the gas emissivity of the molding sand carried out on SIBELCO (S) sand with various binders in alkali-phenolic process for the constant resin content of 1.2 wt.% and 25% of hardener.

Figure 7 presents the gas emissivity of the molding sands. As was found on the basis of the thermal analysis (Figure 2), the thermal destruction of the binders is similar. For this reason, the gas emissivity test, carried out for the sand with the same binder, showed similar gas emissivity features and level.

An important aspect of molding sand preparation is to use a sand matrix of a particular grain size. The larger the casting (larger mold, therefore higher gas emission due to metal-mold contact), the coarser the fraction of sand matrix necessary to ensure proper permeability. Therefore, a comparative study was performed for the sand composition with the analyzed binding materials and another sand with different grain size parameters. Its grain size, given by parameter d_L_, was higher than that for SIBELCO (S) sand.

**Figure 8:**
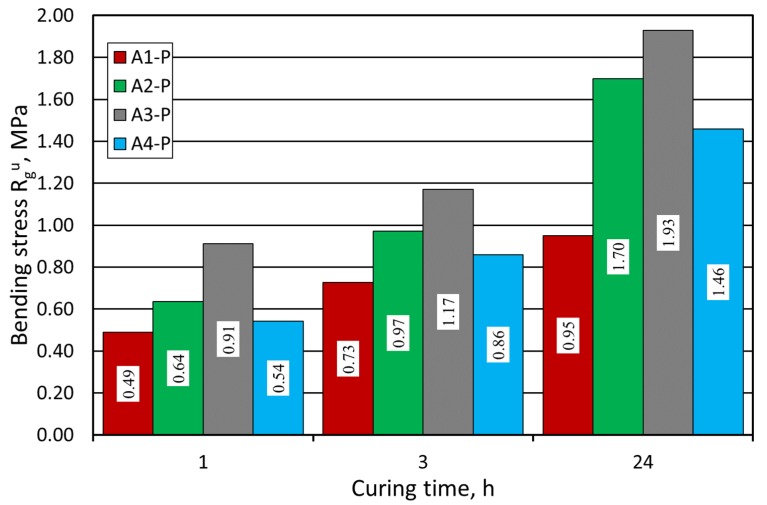
R^u^_g_ bending stress test of the molding sand carried out on Pioma Foundry (P) grain matrix.

Figure 8 presents the results of R^u^_g_ bending stress test of the molding sand, performed on the sand matrix utilized by Pioma Foundry (P). As in the case of the previous grain matrix, the strength relations between used binders were maintained. In the end, after 24 h, all the tensile strength values were higher, particularly for the binders labelled A3 and A2.

**Figure 9:**
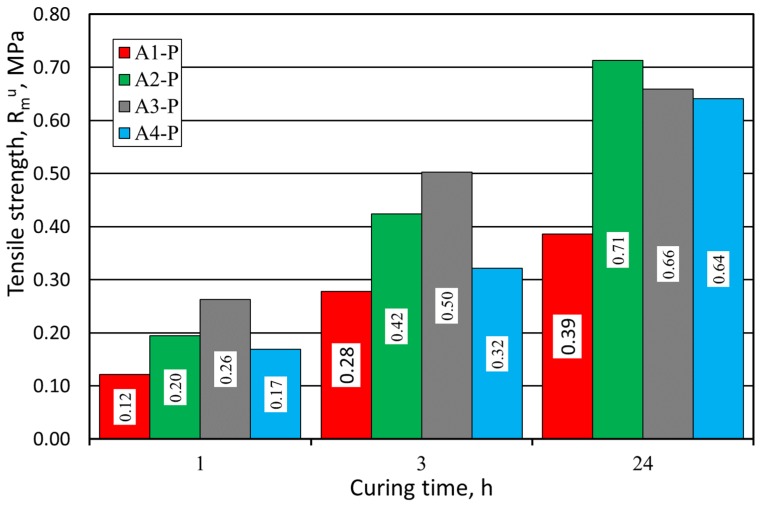
Molding sand R^u^_m_ tensile strength test carried out on Pioma Foundry (P) grain matrix.

The next analyzed parameter was R^u^_m_ tensile strength. Figure 9 shows the results of these tests. The strength properties obtained for the sand from the Pioma Foundry (P) were similar to those obtained for the SIBELCO (S) grain matrix.

**Figure 10:**
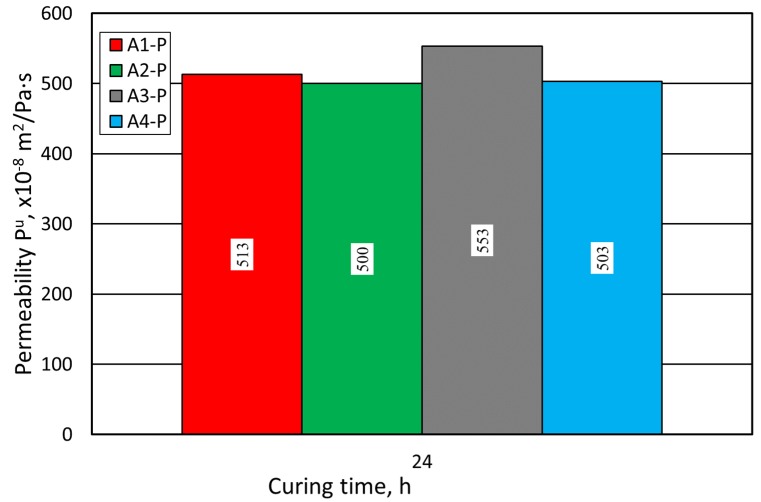
Molding sand P^u^ permeability test carried out on Pioma Foundry (P) grain matrix.

On the other hand, the use of a coarser grain matrix (the sand from Pioma Foundry) with the same amount of added binding material provided greater permeability, as compared to the finer SIBELCO (S) sand (Figure 10).

**Figure 11:**
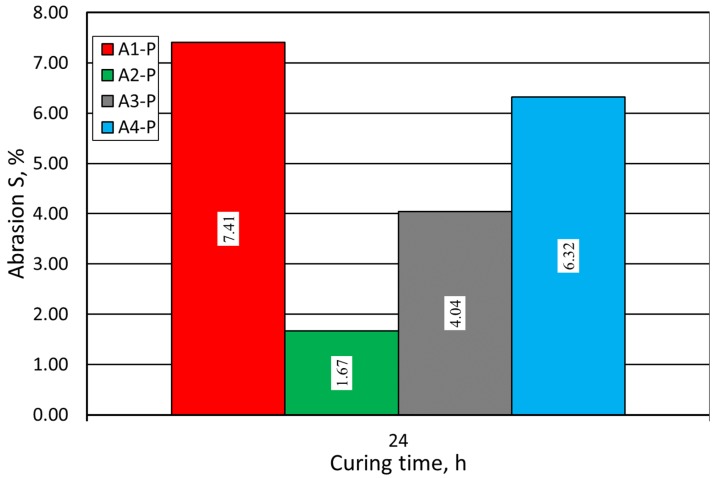
Molding sand S abrasion test carried out on Pioma Foundry (P) grain matrix.

Use of the coarser grain matrix affected the abrasion process of the analyzed molding sands. Larger grains, torn out of the cylindrical sample, strongly affected the final S abrasion parameter for the analyzed sands. So, for the molding sands made on the base of Pioma Foundry sand, the abrasion test results were worse, which is illustrated in Figure 11. As in case of sand with the SIBELCO (S) matrix, the best results were achieved when A2 binder was used.

**Figure 12:**
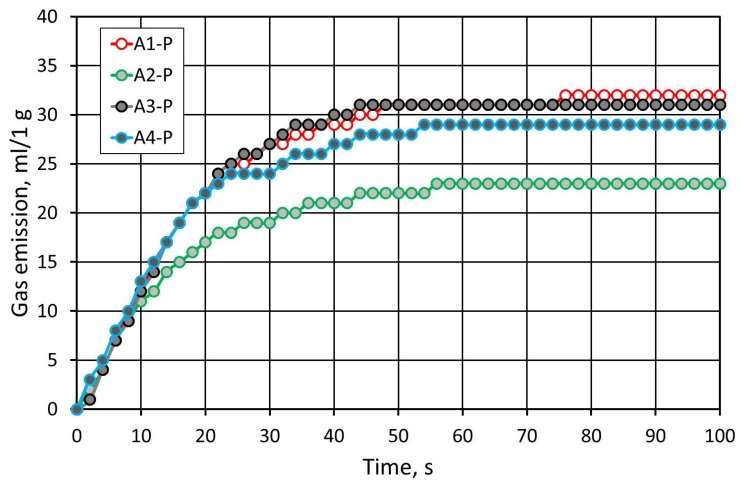
Comparison of the gas emissivity of the molding sand made on the Pioma Foundry (P) sand with various binders in the alkali-phenolic process for the constant resin content of 1.2 wt.% and 25% of hardener.

Figure 12 shows the results of the gas emissivity test of masses carried out on the grain matrix of Pioma Foundry. A slightly smaller gas emission was reported, which was caused by a smaller specific surface area of the sand grains. Additionally, when compared to SIBELCO (S) sand, more diverse emissivity values were observed. The lowest gas emission was recorded for the sand with A2 binder.

## 5. Summary and Conclusions

This article presents the results of the examination of molding sands with alkali-phenolic resin binders. The experiments were carried out for four different binders with the same resin and curing agent proportions and two different sand matrices. The aim of the research was to point to the binder that offered the best alternative for a foundry as a replacement for the currently used furfuryl resin.

Thermal analysis showed a similar thermal decay of the analyzed binding reagents. On the thermogram, favorable features of the multistage degradation and destruction process of these resins may be observed. No analyses of the generated gases were conducted, but, according to the literature and the authors’ own experience, the composition and volume of gases is acceptable even when the highest EU standards are applied. Therefore, from this point of view, furan resin can be replaced with the alkali-phenolic one, especially when the foundry is equipped with efficient systems for removal and treatment of process gasses.

The examination of the strength properties of prepared sands with application of two various grain matrices showed that the sands with A3 and A2 binders demonstrated the best parameters. The higher the strength of a sand with an alkali-phenolic binder, the more predisposed it is to be used as a replacement, taking into account that the previously used sand with furfuryl resin featured greater strength. However, the higher the strength, the bigger the problems with castings’ knock-out. Hence, the strength should always be considered in combination with knock-out ability. From this point of view, the Alphaset system looks quite promising.

The type of the binder used did not affect the sand permeability, as this parameter depends on the grain size of the sand matrix. The abrasion resistance depends on both the binder used and the grain size of the matrix. The lowest abrasion resistance was reported for binders A2 and A3 for the finer of the sands used.

Considering the above, resins labelled A2 and A3 are the recommended binders for the Pioma Foundry, if the company plans to switch from a furan system into an alkali-phenolic binding system.

As mentioned in the Introduction, one of the important parameters of molding sand is its reclaimability—the ability to reuse the sand matrix after the reclamation process, removing the used binder. Thus, future experiments will focus on the reclamation of the chosen sand-resin-hardener systems, to finally convince the foundry industry that the Alphaset system may successfully replace the currently used furan one.

## Figures and Tables

**Table 1:** Molding sand mixtures for the alkali-phenolic process.

Sand Label	Resin	Hardener	Sand Matrix [wt.%]	Resin Ratio [wt.%]	Hardener Ratio [% of the Resin wt.]
MS1	Estrofen (A1)	PR (1)	100	1.2	25
MS2	Sinotherm 8255 (A2)	Aktivator J 120 (2)	100	1.2	25
MS3	Fenotec 280ES (A3)	Fenotec HC Esters HC 30 (3)	100	1.2	25
MS4	Permabind 440 (A4)	Permabind Plus 7 Hardener (4)	100	1.2	25
